# Metabolic Response to *Klebsiella pneumoniae* Infection in an Experimental Rat Model

**DOI:** 10.1371/journal.pone.0051060

**Published:** 2012-11-30

**Authors:** Fangcong Dong, Bin Wang, Lulu Zhang, Huiru Tang, Jieshou Li, Yulan Wang

**Affiliations:** 1 Key Laboratory of Magnetic Resonance in Biological Systems, State Key Laboratory of Magnetic Resonance and Atomic and Molecular Physics, Wuhan Centre for Magnetic Resonance, Wuhan Institute of Physics and Mathematics, Chinese Academy of Sciences, Wuhan, P. R. China; 2 Research Institute of General Surgery, Jinling Hospital, Nanjing, Jiangsu, People’s Republic of China; 3 Graduate School of Chinese Academy of Sciences, Beijing, People’s Republic of China; Fundação Oswaldo Cruz, Brazil

## Abstract

Bacteremia, the presence of viable bacteria in the blood stream, is often associated with several clinical conditions. Bacteremia can lead to multiple organ failure if managed incorrectly, which makes providing suitable nutritional support vital for reducing bacteremia-associated mortality. In order to provide such information, we investigated the metabolic consequences of a *Klebsiella pneumoniae* (*K. pneumoniae*) infection *in vivo* by employing a combination of ^1^H nuclear magnetic resonance spectroscopy and multivariate data analysis. *K. pneumoniae* was intravenously infused in rats; urine and plasma samples were collected at different time intervals. We found that *K. pneumoniae-*induced bacteremia stimulated glycolysis and the tricarboxylic acid cycle and also promoted oxidation of fatty acids and creatine phosphate to facilitate the energy-demanding host response. In addition, *K. pneumoniae* bacteremia also induced anti-endotoxin, anti-inflammatory and anti-oxidization responses in the host. Furthermore, bacteremia could cause a disturbance in the gut microbiotal functions as suggested by alterations in a range of amines and bacteria-host co-metabolites. Our results suggest that supplementation with glucose and a high-fat and choline-rich diet could ameliorate the burdens associated with bacteremia. Our research provides underlying pathological processes of bacteremia and a better understanding of the clinical and biochemical manifestations of bacteremia.

## Introduction

Bacteremia is the presence of viable bacteria in the bloodstream and is the consequence of several clinical conditions, such as trauma, burn injury, abdominal surgery, and catheterization [1]–[3]. The spread of bacteria to the bloodstream leads to a hyperactive inflammatory immune response and subsequent production of excessive inflammatory cytokines, resulting in a systemic inflammatory response syndrome and multiple organ dysfunctions [4]–[5]. *Klebsiella pneumoniae* (*K. pneumoniae*) is a facultative anaerobic gram-negative *bacilli* bacterium and, after *Escherichia coli*, is the second most common cause of community- and hospital-acquired bacteria [6]–[8]. Incidence and mortality rates associated with bacteremia are 7.1 in 100,000 per year and 1.3 in 100,000 per year, respectively [9]. Nutritional support is important in the management of patients with bacteremia. Previous studies have shown that glutamine treatment decreases the incidence of gram-negative bacteremia and a choline-rich diet improves the survival from endotoxin shock in a rat model [10]–[11]. Bacteremia is expected to generate measurable changes in metabolic levels. Therefore it is possible to monitor dynamic metabolic changes associated with bacteremia and identify metabolites related to the event. Developing an in-depth and systematic study of changes associated with bacteremia could provide a comprehensive view on the host metabolic response to bacteremia and open a window for nutritional intervention against the disease.

Metabonomics involves multivariate statistical analyses on spectroscopic fingerprints of biofluids generated from nuclear magnetic resonance (NMR) spectroscopy or mass spectrometry [12]–[13]. This is an emerging field of post-genomic science, which has been established as an extremely powerful analytical tool and has widespread applications in diverse research areas including genetics [14], toxicology [15], metabolic regulation [16]–[17], and infectious diseases [18]–[19].

In the current study, we employ ^1^H NMR spectroscopy in conjunction with multivariate data analysis to investigate metabolic changes in response to *K. pneumoniae in vivo.* The aim of the investigation is to uncover the mechanisms of *K. pneumoniae* infection at the metabolic level and to exploit the potential of metabonomics as a guidance tool for the management of bacteremia, which could be important for the improvement of disease survival.

## Materials and Methods

### Bacteria

*K. pneumoniae*, isolated from mesenteric lymph node in rat that suffered from intestinal ischemia and reperfusion injury, was cultured with Luria-Bertani broth (Oxoid Limited, Basingstoke, Hampshire, England) for 16 h to stationary phase, producing a concentration of 4×10^10^ colony forming units per mL (CFU/mL). Counting of bacteria was conducted by culturing diluted bacteria on Luria-Bertani agar plates and colonies were counted after 24 hours. Bacterial suspensions were centrifuged at 6000g for 10 min, washed twice and re-suspended in sterile saline solution for infection experiments.

### Chemicals

Sodium chloride, K_2_HPO_4_·3H_2_O, and NaH_2_PO_4_·2H_2_O (analytical grade) were obtained from Guoyao Chemical Co. Ltd. (Shanghai, China). Sodium 3-trimethylsilyl [2,2,3,3-d_4_] propionate (TSP-d_4_) and D_2_O (99.9% in D) were purchased from Cambridge Isotope Laboratories (Miami, FL).

### Ethics Statement

Animal experimental procedures were performed according to the National Guidelines for Experimental Animal Welfare (Ministry of Science and Technology of People’s Republic of China, 2006) and approved by the Animal Welfare Committee of Wuhan Institute of Physics and Mathematics, Chinese Academy of Sciences, with permission from China Hubei Provincial Science and Technology Department. All surgery was performed under isoflurane anesthesia, and all efforts were made to minimize suffering.

### Animal Experiments and Sample Collection

All animals used in this investigation are female Sprague Dawley (SD) rats (120–150 g, 5 weeks old, No. hnaslkj20101332) that were purchased from Hunan Slac Jingda Laboratory Animal Co. Ltd. (Changsha, China), and housed in groups of four at a certified local animal experimental laboratory (No. 00018445) with a 12 h light/dark cycle at a constant temperature of 23±1°C. Animals were allowed to have access to food and water *ad libitum*.

A preliminary experiment was conducted to certify the dosage and duration of infection; the result suggested that 0.3 mL of 4×10^10^ CFU/mL *K. pneumoniae* was the maximum level that could be intravenously injected without causing mortality. In order to follow the infection and recovery processes clinically, 24 SD rats were injected with 0.3 mL of *K. pneumoniae* (4×10^10^ CFU/mL) via the tail and 4 rats were sacrificed at each of the following time points: 4 h, 8 h, 1 day, 2 day, 3 day and 7 day postinfection. Another 8 rats were kept as controls and injected with 0.3 mL of saline solution; they were sacrificed at 4 h after injection. A total of 0.5 mL of whole blood was collected and cultured to measure bacterial burden, plasma samples were also collected in tubes containing ethylene diamine tetra-acetic acid for a white blood cell count as well as C-reactive protein and procalcitonin assays.

A separate animal experiment was conducted for the metabonomics investigation. A total of 24 SD rats were randomly divided into two groups after two weeks of acclimatization. They were subjected to treatments for 14 days: a control group (n = 12) and infection group (n = 12) were intravenously injected with 0.3 ml of sterile saline (0.9% sodium chloride) and 0.3 ml of *K. pneumoniae* (4×10^10 ^CFU/mL) via the tail respectively. Blood and urine samples were collected at 9 time points: before the injection (hour 0), and at 4 h, 8 h, 24 h, 48 h, 3 d, 7 d, 10 d and 14 d postinfection. Urine samples were collected by placing rats individually into empty cages covered with a disposable plastic wrap. Urine was immediately transferred into 1.5 mL Eppendorf tubes, and snapped frozen in liquid nitrogen as soon as rats released a few drops of urine. Between 50 and 60 µL of blood was collected into 0.5 mL Eppendorf tubes containing 10 µL sodium heparin from the tail of the rats by cutting off its tip. Plasma was obtained by centrifugation (Microcentrifuge Hettich MIKRO22 Zentrifugen, Germany) at 4000 g for 10 min. The plasma was then transferred into 0.5 mL Eppendorf tubes, and snapped frozen in liquid nitrogen. Plasma and urine samples were stored in a freezer at −80°C for later analysis. At the end of experimental period (day 15), all animals were sacrificed by cervical dislocation under isoflurane anesthesia after 12 h fasting. No further samples were collected.

### Sample Preparation for NMR Spectroscopy

Plasma samples were prepared by mixing 30 µL plasma with 30 µL saline solution containing 100% D_2_O for the magnetic field lock and the 60 µL sample was transferred into 1.7 mm micro NMR tubes. ^1^H NMR spectra of plasma were recorded at 298 K on a Bruker Avance II 500 MHz NMR spectrometer (Bruker, Germany), equipped with a Bruker 5 mm BBI probe with inverse detection, operating at 500.13 MHz proton frequency. A one-dimensional ^1^H NMR spectra with water presaturation were acquired with Carr-Purcell-Meiboom-Gill (CPMG) pulse sequence [recycle delay −90°-(τ-180°-τ)_n_-acquisition] to attenuate NMR signals from macromolecules. A total transverse relaxation delay (2nτ) of 70 ms was used. 90° pulse was set to about 10.0 µs and 256 transients were collected into 32 K data points for each spectrum with a spectral width of 20 ppm. An anomeric proton signal of α-glucose (δ 5.233) was used as a chemical shift reference.

A total of 550 µL urine sample was mixed with 55 µL phosphate buffer (K_2_HPO_4_/NaH_2_PO_4_, 1.5 M, pH 7.4, 100% D_2_O) containing 0.05% TSP-d_4_ for chemical shift calibration and 0.1% of NaN_3_ for prevention of bacterial contamination [20]. After centrifugation at 12000 *g* for 10 min, the supernatant was transferred into 5 mm NMR tubes for NMR analysis. ^1^H NMR spectra of urine were acquired at 298 K on a Bruker Avance 600 MHz NMR spectrometer equipped with a 5 mm TCI cryogenic probe, with inverse detection using a water presaturation pulse sequence [recycle delay-90°-t_1_-90°-t_m_-90°-acquisition]. The recycle delay was set to 2 s, t_1_ to 3 µs and mixing time (t_m_) to 80 ms. A total of 64 transients for urine spectra were collected. The spectra were referenced to TSP-d_4_ at δ 0.00.

For spectral assignment purposes, a series of two-dimensional NMR spectra were acquired on selected plasma and urine samples, which include ^1^H-^1^H correlation spectroscopy, ^1^H-^1^H total correlation spectroscopy, ^1^H-^13^C heteronuclear single quantum correlation spectroscopy, and ^1^H-^13^C heteronuclear multiple bond correlation spectroscopy. The standard parameters used for these spectral acquisitions have previously been reported [20]–[21].

### NMR Data Processing and Multivariate Data Analysis

All free induction decays were multiplied by an exponential function with a 1 Hz line broadening factor prior to Fourier transformation and all the ^1^H NMR spectra were corrected manually for phase and baseline distortions. The spectral region δ 0.5–9.5 was integrated into regions with an equal width of 0.004 ppm (2 Hz) using an AMIX software package (V2.1, Bruker Biospin, Germany). Regions distorted by imperfect water saturation were discarded together with the regions containing urea signals. These regions are δ 4.5–5.0 for plasma and δ 4.4–6.2 for urine. Each bucketed region was then normalized by probabilistic quotient normalization prior to statistical data analysis [22].

Multivariate data analysis was carried out with the SIMCA-P^+^ software (version 11.0, Umetrics, Sweden). Principal component analysis (PCA) was initially carried out on mean-centered NMR data to generate an overview. Projection to latent structure with discriminant analysis (PLS-DA) and orthogonal projection to latent structure with discriminant analysis (O-PLS-DA) were subsequently conducted with the data scaled to unit variance. The quality of the models was assessed by model parameters; Q^2^, indicated the predictability of the model and R^2^ denoted the interpretability of the model. A 7-fold cross-validation method, permutation test and ANOVA of the cross-validated residuals (CV-ANOVA) test were used to validate the models [23]–[24]. The loadings that indicated altered metabolites after the infection were back-transformed and plotted with a color-coded correlation coefficient for each data point using an in-house developed Matlab script (MATLAB 7.1, the Mathworks Inc., Natwick, USA); this facilitated the interpretation of the results [25]. The color-coded correlation coefficient indicates the importance of the metabolite in contributing to the class separation; a “hot” color (e.g. red) being more important than a “cold” color (e.g. blue). The number of animals used was 12; according to Pearson linear correlation coefficients, a correlation coefficient |r| greater than 0.553 was considered to be significant at p<0.05.

## Results

### Bacteremia and Clinical Biochemistry

In order to establish bacteremia and monitor the development and recovery of bacteremia, bacterial burden, white blood cell count, C-reactive protein and procalcitonin levels in blood were measured at each time point (Table 1). Bacteria were detected at 4 h postinfection, reached its highest levels at 8 h and diminished after 2 days postinfection. The level of procalcitonin followed a similar trend to the bacterial load, although the level of procalcitonin was highest at 1 day postinfection. The white blood cell count was significantly reduced at 4 h postinfection and increased at 7 days postinfection, whilst the level of C-reactive protein was significantly increased at 1 day postinfection.

**Table 1:** Bacterial counts, procalcitonin, white blood cell count and C-reactive protein in blood stream obtained from *K.* *pneumoniae-*infected rats compared to controls^a^.

Time points	Bacterial counts (CFU/mL)	Procalcitonin (pg/mL)	White blood cell count (10^9^ cells/L)	C-reactive protein (mg/L)
h0	0 (0–0)	736±159	9.79±1.56	2.60±2.00
h4	273 (14–660)**	1100±269**	2.70±1.90*	1.80±2.10
h8	801 (188–2400)**	1069±143**	7.65±1.58	0.25±0.44
h24	2 (0–580)*	3477±478**	9.33±1.31	9.55±3.70**
d2	44 (40–50)**	1097±281**	10.05±1.09	<0.01
d3	0 (0–128)	825±76	9.83±1.21	0.60±0.40
d7	0 (0–0)	948±146*	14.64±1.19**	1.20±0.90

a Bacterial counts data are represented as median (range); Procalcitonin, white blood cell count and C-reactive protein are represented as mean ± SD. *p<0.05, **p<0.01.

### Metabolites Assignments with ^1^H NMR Spectroscopy

**Figure 1:**
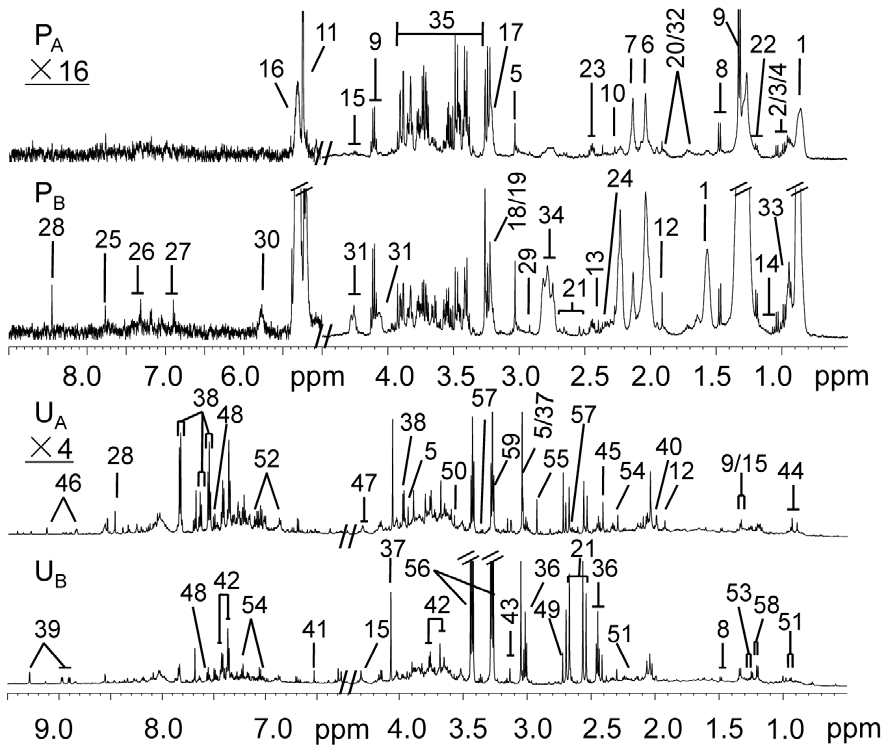
^1^H NMR spectra of plasma and urine from control and *K. pneumoniae* infected rats for 8 hours. Typical 500 MHz ^1^H (CPMG) NMR spectra of plasma obtained from a non-infected SD rat (P_A_) and a rat infected with *K. pneumoniae* for 8 hours (P_B_). The region of δ 5.0–9.0 in the blood plasma spectra was vertically expanded 16 times compared with the region of δ 0.5–4.5; Representative 600 MHZ ^1^H NMR spectra of urine samples obtained from a non-infected SD rat (U_A_) and a rat infected with *K. pneumoniae* for 8 hours (U_B_). The spectral region, δ 6.2–9.5, was vertically expanded 4 times compared with the region of δ 0.5–4.4. Key: 1,lipoprotein; 2,valine; 3,leucine; 4,isoleucine; 5,creatine; 6,*N*-acetyl glycoprotein; 7,*O*-acetyl glycoprotein; 8,alanine; 9,lactate; 10,acetoacetate; 11,α-glucose; 12,acetate; 13,pyruvate; 14,dihydrothymine; 15,threonine; 16,unsaturated fatty acid; 17,choline; 18,phosphorylcholine; 19,glycerophosphocholine; 20,lysine; 21,citrate; 22,_D_-3-hydroxybutyrate; 23,glutamine; 24,glutamate; 25,histidine; 26,phenylalanine; 27,tyrosine; 28,formate; 29,trimethylamine; 30,urea; 31,triglyceride; 32,arginine; 33,ω-3 fatty acid; 34,poly unsaturated fatty acid; 35,glucose and amino acids α-CH resonances; 36,2-oxoglutarate; 37,creatinine; 38,hippurate; 39,1-methylnicotimamide; 40,acetamide; 41,fumarate; 42,phenylacetylglycine; 43,cis-aconitate; 44,pantothenic acid; 45,succinate; 46,*N*-methylnicotinate; 47,malate; 48,indoxyl sulfate; 49,dimethylamine; 50,glycine; 51,isovalerate; 52,2-(4-hydroxyphenyl)propanoic acid; 53,2,3-dihydroxybutyrate; 54,4-cresol glucuronide; 55,dimethylglycine; 56,taurine; 57,hypotaurine; 58,4-deoxyerythronate; 59,trimethylamine *N*-oxide.

Typical ^1^H NMR spectra of blood plasma and urine obtained from both control and *K. pneumoniae* infected rats at 8 hours after treatment were shown in Figure 1. The metabolite resonances were assigned according to literature and 2D NMR spectra. Plasma spectra displayed signals from lipoproteins, unsaturated fatty acid (UFA), poly unsaturated fatty acid (PUFA), ω-3 fatty acid, triglyceride (TG), *N*-acetyl glycoprotein (NAG), *O*-acetyl glycoprotein (OAG), glucose, amino acids, dihydrothymine, carboxylic acids, such as lactate and _D_-3-hydroxybutyrate (3-HB), and choline metabolites. Urine spectra were comprised of tricarboxylic acid (TCA) intermediate metabolites (citrate, 2-oxoglutarate, succinate, fumarate, malate), alanine, taurine, hypotaurine, dimethylglycine (DMG), dimethylamine (DMA), creatinine, pantothenic acid, 4-cresol glucuronide (4-CG), 2,3-dihydroxybutyrate, 4-deoxyerythronate, trimethylamine *N*-oxide (TMAO), 1-methylnicotimamide, and gut microbial-host co-metabolites (hippurate, indoxyl sulfate, and phenylacetylglycine). The detailed NMR assignment can be found in Table S1. To extract the detailed information about *K. pneumoniae-*infected metabolic alterations, multivariate data analysis of these NMR profiles was performed.

### Infection Progression

**Figure 2:**
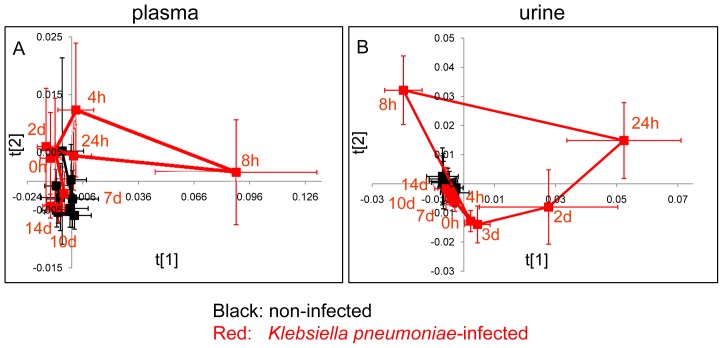
Trajectories of plasma and urinary metabolic profiles of the control group and the infected group at different time intervals. Time-dependent trajectories of plasma (A, R^2^X = 0.928, Q^2^ = 0.918) and urinary (B, R^2^X = 0.789, Q^2^ = 0.614) metabolic profiles of the control group (black squares) and the infection group (red squares) from hour 0 to day 14. Bars denote the standard deviations of each group.

In order to characterize the evolution of the infection through time, PCA was conducted on the NMR data of urine and plasma separately from control and infected rats at all time points. The PCA trajectory plots illustrated the time dependence of the alterations of the plasma and urinary metabolic profiles induced by *K. pneumoniae* infection (Figure 2). Clearly, the global metabolic responses from the profiles of plasma showed a rapid metabolic shift at 8 h postinfection and a speedy recovery through time; this is in contrast to the trajectory of urine profiles, where gradual recovery appears to be made. Cross-validated PLS-DA pair wise comparisons between spectra obtained from the control group and infection group were constructed and validated by a permutation test; it suggested that metabolic disturbances in plasma were diminished at day 10 postinfection while metabolic deviations in urine could still be observed even at day 14 postinfection (Table 2).

**Table 2:** O-PLS-DA Cross-validation Model Summary for Pair-wise Comparison between NMR Spectra of Plasma and Urine Obtained from *K. pneumoniae-*infected Rats Compared to Controls on Different Time Points^a^.

	R^2^X(Q^2^)								
	h0	h4	h8	h24	d2	d3	d7	d10	d14
**Plasma**	0.18 (0.37)	0.23 (0.70)	0.39 (0.88)	0.29 (0.90)	0.32 (0.93)	0.26 (0.87)	0.18 (0.60)	0.24 (0.53)	0.17 (0.42)
**Permutation tests**	×	√	√	√	√	√	√	×	×
**P-value** b	7.18e–02	8.67e–05	2.43e–08	3.55e–09	9.00e–11	3.16e–08	1.04e–03	4.52e–03	2.86e–02
**Urine**	0.26 (–0.14)	0.33 (0.85)	0.42 (0.95)	0.42 (0.89)	0.45 (0.90)	0.36 (0.82)	0.32 (0.55)	0.33 (0.51)	0.24 (0.63)
**Permutation tests**	×	√	√	√	√	√	√	√	√
**P-value**	1	1.58e–06	4.69e–12	6.42e–09	3.79e–09	8.55e–07	4.42e–03	6.63e–03	6.00e–04

a Values are cumulative. One PLS component and one orthogonal component are calculated. The R^2^X value shows how much variation in the data set is explained by the model. The Q^2^ value represents the predictability of the model.

b All models have been validated using permutation tests (n = 200) and ANOVA of the cross-validated residuals (CV-ANOVA) tests. P-values are obtained from CV-ANOVA tests. The underlined values indicate valid models (p<0.05).

### Metabolic Changes in Plasma Samples

**Figure 3:**
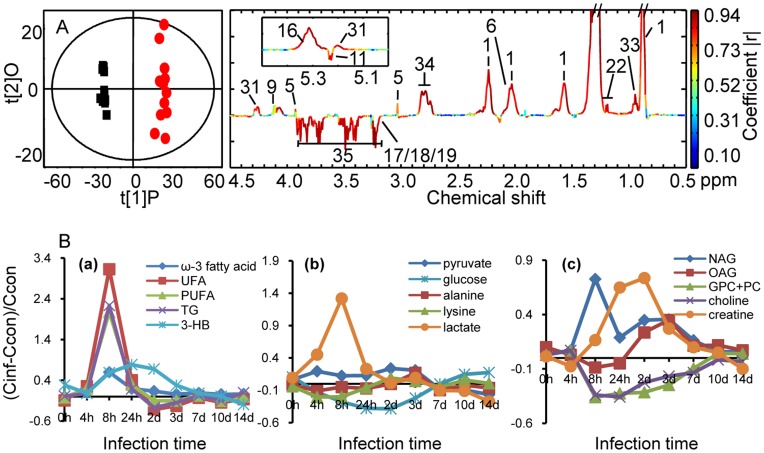
O-PLS-DA comparison between plasma spectra from *K. pneumoniae* infected rats and corresponding controls and metabolite concentration changes relative to corresponding controls at different time points after *K. pneumoniae* infection. (A) Cross validated O-PLS-DA scores (left hand side) and coefficient plots (right hand side) generated from NMR spectral data of plasma of rats at 8 hours after *K. pneumoniae* infection (red dots), compared with those of non-infected (black squares). (B) a-c plots show metabolites changes in plasma. C_inf_ and C_con_ stand for the averaged concentration in the infection and control group, respectively.

To identify the metabolites altered after the infection, the O-PLS-DA models comparing the control group and infection group were constructed for plasma profiles. CV-ANOVA validated model parameters (R^2^, Q^2^ and p values) are listed in Table 2. For illustrative purpose, we only showed the cross-validated scores plot and corresponding coefficient plot generated from the model constructed for 8 h after infection (Figure 3A). The time dependence of metabolic alterations was displayed in Figure 3B. Compared with the control rats, *K. pneumoniae-*infected rats produce significantly higher levels of lipoproteins, TG, UFA, PUFA, ω-3 fatty acid, 3-HB, lactate, NAG and creatine, and lower levels of glucose and membrane related metabolites such as choline, phosphorylcholine (PC), and glycerophosphocholine (GPC) in plasma.

### Metabolic Changes in Urine Samples

**Figure 4:**
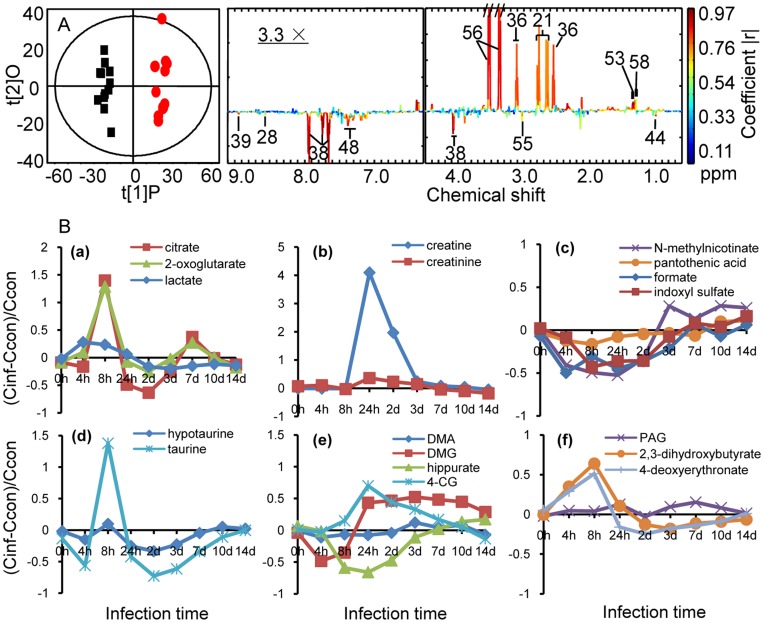
O-PLS-DA comparison between urine spectra from *K. pneumoniae* infected rats and corresponding controls and metabolite concentration changes relative to corresponding controls at different time points after *K. pneumoniae* infection. (A) Cross validated O-PLS-DA scores (left hand side) and coefficient plots (right hand side) generated from NMR spectral data of urine of rats at 8 hours after *K. pneumoniae* infection (red dots), compared with those of non-infected (black squares). (B) a-f plots show metabolites changes in urine. C_inf_ and C_con_ stand for the averaged concentration in the infection and control group, respectively.

Similar analysis was performed for urinary profiles and CV-ANOVA validated model parameters (R^2^, Q^2^ and p values) are also listed in Table 2. The cross-validated scores plot and corresponding coefficient plot generated for urine profiles at 8 h after infection is displayed in Figure 4A. The time dependence of urinary metabolic alterations was displayed in Figure 4B. A range of urinary metabolites were also altered after *K. pneumoniae-*infection. The levels of creatine were elevated markedly at 24h post infection and leveled off at 3 days post infection. The levels of taurine, citrate, 2-oxoglutarate, 2,3-dihydroxybutyrate, 4-deoxyerythronate and hypotaurine altered concurrently; these displayed an initial increase to the maximum level at 8 h postinfection and decrease at 2 days postinfection. In contrast, the levels of hippurate, DMG, DMA, *N*-methylnicotinate, formate and indoxyl sulfate and pantothenic acid were reduced at the early stage of infection and gradually increased at the later stage of the infection. Unlike metabolites in plasma, full recovery of urinary metabolites was not achieved after 14 days postinfection.

## Discussion

Bacteremia is caused by bacterial infection in the blood and can rapidly spread to other parts of the body, causing multiple organ failure. In order to understand metabolic perturbation associated with bacteremia and thus provide a useful nutritional guide for patients with bacteremia, we employed a rat model to investigate metabolic modification induced by *K. pneumoniae* infection, using a metabonomic strategy.

### Infection Progression

PCA trajectory of plasma profiles illustrated relocations between 4 h and 24 h postinfection with maximum deviations at 8 h postinfection, which matched perfectly with the bacterial burden in bloodstream (Table 1) which displays plasma profile as a better indication for bacteremia than other immunological response parameters (such as white blood cells, C-reactive protein and procalcitonin). Inspection of concurrently altered metabolites (Figure 3B) suggested that sharp elevations in the levels of ω-3 fatty acid, UFA, PUFA, TG, lactate and NAG in plasma at 8 h postinfection contributed to the maximum deviations in metabolic space observed at 8 h postinfection. Given that bacterial cultures in blood stream generally takes 24 h, blood tests for aforementioned metabolites could be a valuable and early indicator of bacteremia.

### Energy Metabolism

**Figure 5:**
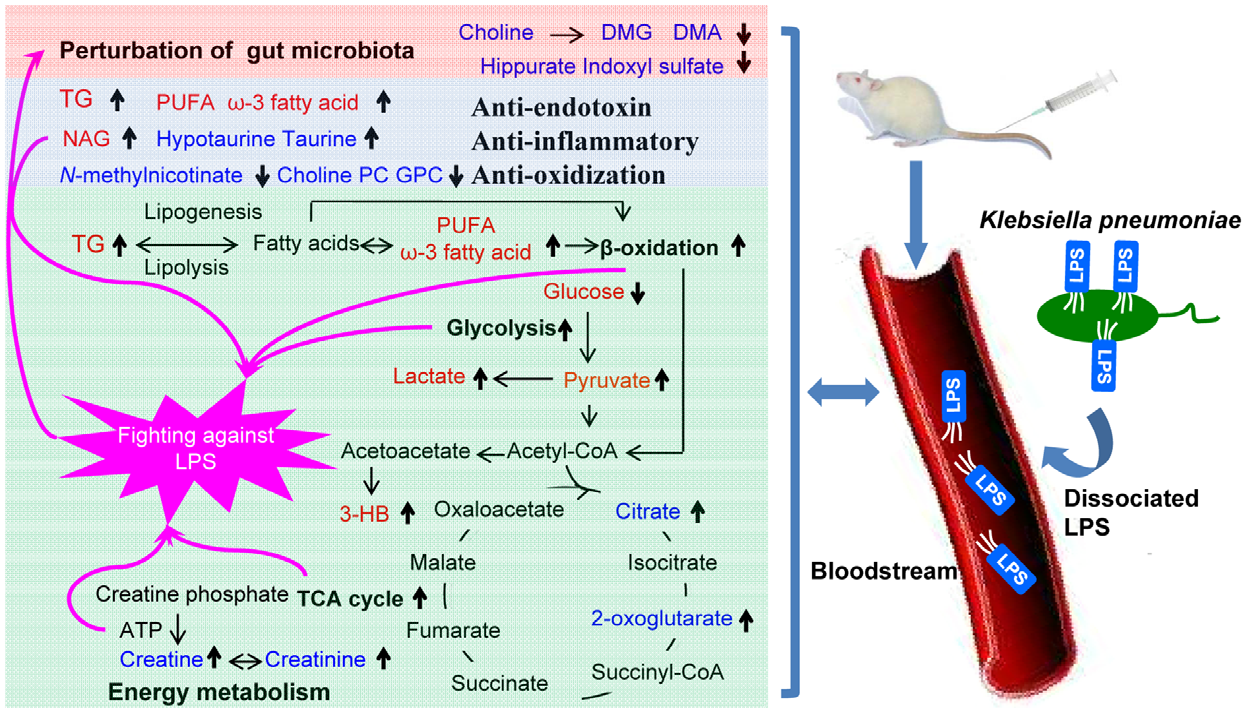
Schematic representation of the metabolites and metabolic pathways in *K. pneumoniae* bacteremia. The metabolites in red indicate the changes in plasma and those in blue indicate the changes in urine whereas those in black were not observed; the arrows pointing up and down denoted relative increase and decrease in the infected group compared with the controls.

We observed a marked reduction in the levels of glucose in plasma of infected rats (Figure 3B). This suggested that stimulated glycolysis is associated with bacteremia; concurrent elevation in the levels of lactate and pyruvate support this notion. The raised urinary level of TCA cycle intermediates in the infected group, such as 2-oxoglutarate and citrate (Figure 4B), suggested that the stimulated glycolysis facilitates the rate of the TCA cycle. Previous metabolic investigation of *Trypanosoma brucei brucei* infection in mice also observed stimulated glycolysis [26]. The stimulated glycolysis and TCA cycle reflect the high energy expenditure that is required to fight the infectious process (Figure 5). This is consistent with a previous report stating that bacteremia is accompanied by a decline of mean arterial blood pressure, hypothermia, leucopenia, and hypoglycemia. Disturbed hepatic glycogen mobilization is likely to partially result in hypoglycemia because excessive burdens bacteria and endotoxin could directly lead to liver injury, which is caused by the liver macrophage acting as a filter to remove bacteria from the bloodstream [27]. Administrating glucose to patients with bacteremia could potentially supply the extra energy required to fight the infection, which could reduce the bacteriemia-associated mortality; this has been previously suggested [28].

One of the most prominent findings in the current study was the increase of TG and lipoproteins in plasma (Figure 3B) after *K. pneumoniae* infection. TGs played an important role in metabolism as an energy source. TGs are constituents of lipoproteins, which deliver the fatty acids to and from adipocytes. When the body requires fatty acids as an energy source, the hormone glucagon signals hormone-sensitive lipase to break down TGs to release free fatty acids. Previous studies have demonstrated that infection and inflammation induce marked changes in lipid and lipoprotein metabolism, including increased serum fatty acids and TGs, increased hepatic TG production and very-low-density lipoprotein secretion and increased adipose tissue lipolysis [29]–[30]. Our observation of a significant increase in the levels of TG and lipoprotein in plasma after *K. pneumoniae* infection suggest that bacteria provoke a dramatic response in the host (Figure 5). Our findings are in good agreement with previously observed results of patients with cholera and patients experiencing polymicrobial infection [31]–[32]. In addition, we observed increased levels of ketone bodies (such as 3-HB in plasma) from the infected rats, suggesting that existence of *K. pneumoniae* in the bloodstream promotes the β-oxidation of fatty acids in mitochondria (Figure 5). The metabolic profiles of the plasma showed a strong increase in the β-oxidation and a drop in glucose concentration which could mirror the high demand of the body for energy in response to bacterial infection. It is known that the oxidation of fatty acid produces more energy per molecule than glycolysis, therefore ATP generated from fatty acid oxidation is an important energy source required by the liver, lung and kidney to function during severe sepsis. The inability to generate energy via fatty acid oxidation might contribute to the development of multiple organ failure. Furthermore, elevation of creatinine was associated with bacteremia (Figure 4). Creatinine and creatine are inter-convertible metabolites. Creatine is generated from the break down of creatine phosphate, an energy reserve in skeletal muscle; ATP is released when there is a high energy demand [33]. The level of creatine in plasma increases in critically ill patients due to the intracellular breakdown of creatine phosphate to creatine and inorganic phosphates, which restores the dwindling supply of ATP [34].

### Anti-endotoxin, Anti-inflammatory and Anti-oxidization Responses

A sharp rise of lipoproteins in the plasma of rats that have been challenged with bacteria could be one of the anti-endotoxin responses by the host (Figure 5). There is substantial evidence showing that triglyceride-rich lipoproteins can bind and neutralize lipopolysaccharide (LPS), a major component of the cell wall of gram-negative bacteria. Lipoprotein-lipopolysaccharide complexes can ameliorate the effects of the host immune defense to bacterial infection [35]. Hence detoxification by lipoproteins prevents endotoxin from initiating an inflammatory response [36]. New evidence shows that a high-fat diet results in increased plasma triacylglycerol and apolipoprotein B levels, and can significantly decrease endotoxemia and bacterial translocation after hemorrhage [37]. Our observation of marked elevation of lipoproteins is consistent with the anti-endotoxin function of lipoproteins [38].

In addition, increased levels of poly unsaturated fatty acid (PUFA) and ω-3 fatty acid were observed simultaneously in plasma of infected rats (Figure 3B). Other studies have shown an increase in the concentrations of PUFA (such as linolenic acid, docosapentaenoic acid and docosahexaenoic acid) in plasma of septic rats [39]. PUFA, principally classified as ω-6 fatty acids and ω-3 fatty acids, have roles in regulating inflammatory responses. The exact roles of ω-6 fatty acids are still unclear. For example, eicosanoids, including prostaglandins, thromboxanes, leukotrienes and other oxidised derivatives, are key mediators and regulators of inflammation; they are mainly synthesized from arachidonica acid, a 20 carbon ω-6 fatty acid [40]–[42], whilst lipoxins, derivatives of ω-6 fatty acids, play important roles in anti-inflammatory processes [43]. ω-3 fatty acids (such as docosahexaenoic acid and eicosapentaenoic acid) was reported to decrease the production of inflammatory eicosanoids (prostaglandin E_2_, thromboxane B_2_, leukotriene B_4_), cytokines, and reactive oxygen species and the expression of adhesion molecules [44]–[45]. Although further investigation is needed to certify the levels and roles of ω-6 fatty acids, the current observed marked increase in ω-3 fatty acid implicates anti-inflammatory effect, particularly at 8 h postinfection. In addition, the NAG in rat plasma is known to represent “acute-phase” glycoprotein in animals under inflammatory conditions and may be useful in the diagnosis and prognosis of acute and chronic inflammatory disorders [46]–[48]. Hence from a metabolism point of view, the observation of elevated levels of NAG and ω-3 fatty acid was in concurrence with the inflammatory response. Anti-inflammatory responses of the host are also manifested in the increased levels of procalcitonin (Table 1) and concurrently the reduced levels of membrane metabolite, phosphocholine. One of the mechanisms of eliminating bacteria is binding C-reactive protein to phosphocholine on the surface of bacteria. The binding may not be specific to phosphocholine on the surface of bacteria as Bach et al has demonstrated by the binding between C-reactive protein isolated from rabbit with phosphocholine *in vitro* [49]. The interactions between C-reactive protein and phosphocholine could in turn explain the reduced levels of phosphocholine observed in the infected rats and the inconsistence between the levels of C-reactive protein and bacterial load (Table 1).

As mentioned previously that bacteremia-induced β-oxidation of lipid, free radicals generated from this β-oxidation would no doubt promote anti-oxidative response from the host (Figure 5). Indeed, here we have observed elevation in the levels of urinary hypotuarine at 8 hours postinfection and its alteration followed the same trend as the levels of fatty acids (Figure 3B and 4B). Promotion of lipid oxidation was previously observed in mice infected with *Trypanosoma brucei brucei* [26]. Hypotaurine is an intermediate of taurine biosynthesis [50], and has been implicated in a wide array of physiological phenomena including membrane stabilization antioxidant, and the regulation of the pro-inflammatory and immune response [51]. In addition, reduction in the levels of *N*-methylnicotinate is associated with bacteremia. *N*-methylnicotinate is the methylated metabolite of niacin (vitamin B_3_) and can be generated during the conversion of *S*-adenosyl-methionine to *S*-adenosyl-homocysteine during cysteine biosynthesis (which an important substrate for glutathione synthesis). Hence a depleted level of *N*-methylnicotinate represents an anti-oxidation response of the host. Interestingly, dietary choline participates in the anti-oxidative processes by enhancing the *S*-adenosyl-methionine to *S*-adenosyl-homocysteine ratio, and regulating the activities of methyltransferases as well as promoting the formation of glutathione. This results in an attenuated inflammatory response, reduced tissue injury and mortality in the rat [52]. Our results suggest that choline supplementation during sepsis could be beneficial to patients.

### Disturbance of Gut Microbes

In our current investigation, decreased levels of choline in plasma, and concurrent decreased levels of DMA and DMG in urine were observed in rats treated with *K. pneumoniae*. Previous research has demonstrated that urinary DMA and DMG are produced via the action of gut microbiota on choline [53]. Therefore, it is plausible to suggest that bacteremia causes a disturbance to gut microbiota (Figure 5). The changes in gut microbial co-metabolites, such as hippurate and indoxyl sulfate further validated our suggestion. Hippurate, generated in the liver, originates from bacterial action upon plant phenols to produce benzoate, which becomes conjugated with glycine. Indoxyl sulfate is the metabolite of tryptophan under the role of a subset of microbiota that has tryptophanase activity. Alterations in the level of hippurate and indoxyl sulfate were previously reported as a consequence of the perturbation in gut microbiota [54]–[55]. However, since no previous report has shown the association between the changes of gut microbiota and bacteremia, further microbiological studies are warranted to ascertain this association.

### Conclusions

In summary, we have characterized time dependence of plasma and urinary metabolic alterations in response to *K. pneumoniae* infection using the metabonomic strategy, indicative of global changes in metabolic regulation. We have shown that metabolic profiles of plasma could be a better indication of bacteremia. *K. pneumoniae* bacteremia disrupts energy metabolism, which is manifested by stimulated glycolysis, TCA cycle and oxidation of lipid and creatine phosphate (Figure 5). In addition, *K. pneumoniae* bacteremia induced focused anti-endotoxin, anti-inflammatory and anti-oxidization responses. Further investigation is needed to validate the disruption of the gut microbiota balance. Our results indicated that infection by *K. pneumoniae* caused altered metabolites that act as a guide for clinical nutrition intake in human conditions of bacteremia. An integrated NMR analysis of plasma and urine provided a holistic method for elucidating metabolic cross-talk between the host and the bacteria *in vivo* during the progress of the infection. Hence, a global metabolic profiling strategy based on ^1^H NMR spectroscopy in conjunction with multivariate data analysis can be utilized for the development of novel, valid, and rapid methods for disease management.

## Supporting Information

Table S1 **^1^H and ^13^C NMR data and assignments of the metabolites in rat plasma and urine.**
(DOC)
