# Supplementary material for: Metabolic Response to Klebsiella pneumoniae Infection in an Experimental Rat Model
Source: PLoS One. 2012 Nov 30;7(11):e51060. doi: 10.1371/journal.pone.0051060 (PMC3511377; doi:10.1371/journal.pone.0051060)
Supplement: Table S1 — 1H and 13C NMR data and assignments of the metabolites in rat plasma and urine. (DOC) [file pone.0051060.s001.doc]

**Supporting Information:**

**Table S1**. ^1^H and ^13^C NMR data and assignments of the metabolites in rat plasma and urine.

| No. | Metabolites | Moieties | δ^1^H (multiplicity)^a^ | δ^13^C | Biofluid^b^ |
| --- | --- | --- | --- | --- | --- |
|  | lipoprotein | CH_3_; CH_2_; CH_2_CH_2_CO;  CH_2_=C; CH_2_CO | 0.89(m); 1.25(m); 1.59(m); 2.02(m); 2.21(m) | c | P |
|  | valine | γCH_3_; γ’CH_3_; βCH; αCH | 0.98(d); 1.04(d); 2.27(m);  3.61(d) | 19.5; 20.9; 31.4; 63.1 | P |
|  | leucine | δCH_3_; δ’CH_3_; γCH; αCH_2_ | 0.95(d); 0.96(d); 1.69(m);  3.73(t) | 24.8; 24.8; 26.9; 42.0 | P |
|  | isoleucine | γCH_3_; δCH_3_; βCH; αCH | 0.93(t); 1.00(d); 1.99(m);  3.68(d) | 13.8; 17.3; 38.5; 62.6 | P |
|  | creatine | CH_2_; CH_3_ | 3.03(s); 3.92(s) | 39.4; 56.7 | P,U |
|  | *N*-acetyl glycoprotein (NAG) | CH_3_ | 2.04(s) | c | P,U |
|  | *O*-acetyl glycoprotein (OAG) | CH_3_ | 2.13(s) | c | P,U |
|  | alanine | βCH_3_; αCH | 1.47(d); 3.78(q) | 19.0; 53.6 | P,U |
|  | lactate | βCH_3_; αCH | 1.33(d); 4.11(q) | 23.4; 71.0 | P,U |
|  | acetoacetate | CH_3_ | 2.27(s) | 32.5 | P |
|  | α-glucose | H1 | 5.23(d) | 94.8 | P |
|  | acetate | CH_3_ | 1.91(s) | 25.8 | P,U |
|  | pyruvate | βCH_3_ | 2.37(s) | 30.3 | P |
|  | dihydrothymine | CH_3_; CH; CH_2_; CH_2_’ | 1.07(d); 2.47(m); 3.52(m);  3.69(m) | 16.5; 47.8; 67.3 | P |
|  | threonine | γCH_3_; αCH; βCH | 1.32(d); 3.58(d); 4.25(m) | 22.9; 63.1; 68.7 | P,U |
|  | unsaturated fatty acid (UFA) | CH=CH | 5.30 | c | P |
|  | choline | N(CH_3_)_3_; N-CH_2_; O-CH_2_ | 3.20(s); 3.52(dd); 4.05(m) | 56.6; 73.9; 58.5 | P |
|  | phosphorylcholine (PC) | CH_3_; N-CH_2_; O-CH_2_ | 3.21(s); 3.63(t); 4.20(m) | 56.6; 65.7; 68.9 | P |
|  | glycerophosphocholine (GPC) | CH_3_; N-CH_2_; O-CH_2_ | 3.21(s); 3.68(m); 4.32(m) | 56.6; 69.3; 68.9 | P |
|  | lysine | γCH_2_; δCH_2_; βCH_2_; εCH_2_; αCH | 1.49(m); 1.70(m); 1.89(m);  3.02(t); 3.76(t) | 24.3; 28.9; 32.4; 41.7; 57.0 | P |
|  | citrate | half CH_2_; half CH_2_ | 2.56(d); 2.67(d) | 42.5 | P,U |
|  | _D_-3-hydroxybutyrate (3-HB) | γCH_3_; αCH_2_; α’CH_2_; βCH | 1.18(d); 2.31(dd); 2.38(dd);  4.16(m) | 24.5; 48.9; 68.3 | P |
|  | glutamine | αCH; βCH_2_; γCH_2_ | 2.13(m); 2.44(m); 3.76(m) | 29.0; 33.8; 57.2 | P |
|  | glutamate | αCH; βCH_2_; γCH_2_ | 2.07(m); 2.33(m); 3.72(m) | 29.5; 36.3; 56.2 | P |
|  | histidine | H4; H2 | 7.08(d); 7.75(d) | 119.0; 139.2 | P |
|  | phenylalanine | H2 & H6; H4; H3 & H5 | 7.31(m); 7.37(m); 7.42(m) | 131.9; 131.2; 131.8 | P |
|  | tyrosine | CH; CH | 6.89(d); 7.18(d) | 118.7; 133.4 | P,U |
|  | formate | H-COOH | 8.45(s) | 151.8 | P,U |
|  | trimethylamine (TMA) | CH_3_ | 2.92(s) | 41.6 | P |
|  | urea | NH_2_ | 5.78(s) | c | P |
|  | triglyceride (TG) | CH_2_OCOR; CH_2_OCOR; CHOCOR | 4.06(m); 4.26(m); 5.20(m) | c | P |
|  | arginine | CH_2_; CH_2_; CH_2_; CH | 1.70(m); 1.93(m); 3.25(t); 3.77(t) | 26.5; 30.7; 43.3; 57.3 | P |
|  | ω-3 fatty acid | CH_3_(CH_2_CH=CH)_n_ | 0.92(m) | c | P |
|  | poly unsaturated fatty acid (PUFA) | C=CCH_2_C=C | 2.78(m) | c | P |
|  | glucose and amino acids | αCH resonances | 3.3-3.9 | c | P |
|  | 2-oxoglutarate | βCH_2_; γCH_2_ | 2.45(t); 3.01(t) | 33.4; 38.4 | U |
|  | creatinine | CH_3_; CH_2_ | 3.05(s); 4.06(s) | 32.9; 59.0 | U |
|  | hippurate | CH_2_; CH; CH; CH; NH | 3.97(d); 7.56(t); 7.65(t);  7.84(d); 8.55(s) | 46.5; 131.9; 135.3; 130.2; 149.8 | U |
|  | 1-methylnicotinamide | CH_3_; H5; H4; H6; H2 | 4.48(s); 8.19(d); 8.90(d);  8.97(d); 9.28(s) | 51.3 | U |
|  | acetamide | CH_3_ | 1.99(s) | 24.6 | U |
|  | fumarate | CH | 6.52(s) | 138.0 | U |
|  | phenylacetylglycine (PAG) | 10-CH; 7-CH; 3,5-CH; 2,6-CH | 3.68(s); 3.76(d); 7.36(m);  7.42(m) | 44.9; 46.1; 132.4; 131.9 | U |
|  | cis-aconitate | CH_2_; CH | 3.12(d); 5.71(s) | 46.3; 127.2 | U |
|  | pantothenic acid | CH_3_; CH_3_; CH_2_; CH_2_; CH_2_; CH | 0.90(s); 0.94(s); 2.42(t);  3.45(t); 3.52(s); 3.99(s); | 21.7; 23.4; 39.8; 38.5; 71.0; 78.5 | U |
|  | succinate | CH_2_ | 2.41(s) | 37.0 | U |
|  | *N*-methylnicotinate | CH_3_; CH; CH; CH | 4.44(s); 8.08(m); 8.84(m);  9.12(s) | 51.2 | U |
|  | malate | CH_2_; CH_2_’; CH | 2.37(dd); 2.67(dd); 4.32(dd) | 44.5; 44.5; 73.3 | U |
|  | indoxyl sulfate | CH; CH; CH; CH | 7.20(dd); 7.28(dd); 7.50(d);  7.71(d) | 122.7; 125.4; 115.0; 120.6 | U |
|  | dimethylamine (DMA) | CH_3_ | 2.72(s) | 37.4 | U |
|  | glycine | αCH | 3.56(s) | 44.4 | P,U |
|  | isovalerate | CH_3_; CH; CH_2_ | 0.94(d); 2.01(m); 2.17(d) | 24.5; 28.3; 48.1 | U |
|  | 2-(4-hydroxyphenyl)propanoic acid | CH_3_; CH; o-CH; m-CH | 1.37(d); 3.58(q); 6.86(m); 7.17(m) | 22.0; 74.3; 118.5; 122.8 | U |
|  | 2,3-dihydroxybutyrate | CH_3_; CH; CH | 1.24(d); 3.42(m); 3.85(d) | 20.8; 69.5; 77.5 | U |
|  | 4-cresol glucuronide (4-CG) | CH_3_; ring-CH; o-CH; m-CH | 2.30(s); 5.08(d); 7.05(d); 7.23(d) | 22.0; 103.2; 119.6; 133.3 | U |
|  | dimethylglycine (DMG) | CH_3_; CH_2_ | 2.93(s); 3.72(s) | 46.2; 62.6 | U |
|  | taurine | -CH_2_-SO_3_; -CH_2_-NH_2_ | 3.27(t); 3.43(t) | 50.5; 38.3 | U |
|  | hypotaurine | -CH_2_-SO_2_; -CH_2_-NH_2_ | 2.66(t); 3.36(t) | 58.7; 36.4 | U |
|  | 4-deoxyerythronate | CH_3_; CH; CH | 1.20(d); 3.65(m); 4.00(d) | 18.8; 70.5; 77.5 | U |
|  | trimethylamine *N*-oxide (TMAO) | CH_3_ | 3.28(s) | 62.1 | U |

^a^s, singlet; d, doubles; t, triples; m, multiplets; q, quartet; dd, double doublet.

^b^P, plasma; U, urine.

^c^ The signals or the multiplicities were not determined.
